# Central retinal vein occlusion in a pediatric patient with SLE and antiphospholipid antibodies without anti-cardiolipin or anti-β2 glycoprotein I antibodies

**DOI:** 10.1186/1471-2431-14-116

**Published:** 2014-05-03

**Authors:** Seigo Korematsu, Hironori Goto, Chika Gotoh, Ryoko Ohki, Toshiaki Kubota, Tatsuro Izumi

**Affiliations:** 1 Department of Pediatrics and Child Neurology, Oita University Faculty of Medicine, Hasama, Yufu, Oita 879-5593, Japan; 2 Department of Ophthalmology, Oita University Faculty of Medicine, Hasama, Yufu, Oita 879-5593, Japan

**Keywords:** Central retinal vein occlusion, Systemic lupus erythematosus, Antiphospholipid antibody syndrome, Anti-phosphatidylcholine antibody

## Abstract

**Background:**

Antiphospholipid antibody syndrome is characterized by venous and/or arterial thrombosis, and is found in patients with systemic lupus erythematosus. Its diagnosis requires the presence of both clinical and laboratory findings, such as positive anti-cardiolipin and anti-β2 glycoprotein I antibodies and lupus anticoagulant. However, cardiolipin is a minor component of the vascular endothelial cells in human, and phosphatidylcholine and phosphatidylethanolamine are major components.

**Case presentation:**

A 15-year-old female suddenly developed massive left intraretinal hemorrhaging due to central retinal vein occlusion. She also had a butterfly rash, and her laboratory findings revealed positive serum anti-nuclear antibodies and decreased serum complement. During this episode, she was diagnosed with systemic lupus erythematosus. Although she was negative for serum anti-cardiolipin IgG and anti-β2 glycoprotein I antibodies as well as lupus anticoagulant, her serum anti-phosphatidylcholine, anti-phosphatidylethanolamine, anti-phosphatidylinositol and phosphatidylserine IgG antibodies levels were increased.

**Conclusion:**

Pediatric cases of central retinal vein occlusion are rare. Even in patients without anti-cardiolipin or anti-β2 glycoprotein I antibodies and lupus anticoagulant, there is the potential for the development of antiphospholipid antibody-related thrombosis.

## Background

Antiphospholipid antibody syndrome (APS) is characterized by venous and/or arterial thrombosis and recurrent fetal loss, and is found in patients with systemic lupus erythematosus (SLE) [1]. A diagnosis of APS requires the presence of both clinical and laboratory findings, such as positive anti-cardiolipin (CL) and anti-β2 glycoprotein I antibodies and lupus anticoagulant. However, CL is a minor component of phospholipids in humans [2].

This report describes central retinal vein occlusion (CRVO) in a pediatric patient with SLE and anti-phospholipids antibodies without anti-CL antibody.

## Case presentation

**Figure 1:**
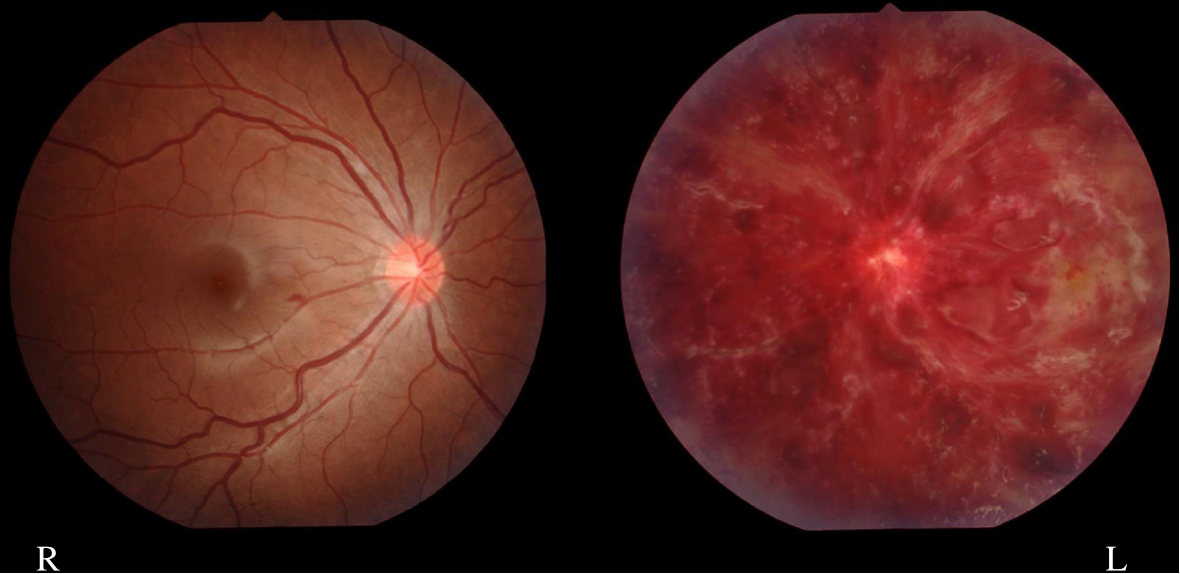
**Finding of eye fundus.** A 15-year-old female patient showed massive left intraretinal hemorrhaging.

A female patient was born without any perinatal problems and her psychomotor and growth development had been normal. Her father had Crohn’s disease and her elder brother had atopic dermatitis. She developed allergic conjunctivitis when she was around 10 years old and was administered betamethasone eye-drops. She also developed a butterfly rash and photosensitivity at the age of 14. And then, she suddenly developed a left visual disturbance without any premonitory sign at the age of 15. Her visual acuity (left: 0.04, right: 1.2) showed asymmetry. Her eye fundus examination showed massive left intraretinal hemorrhaging due to CRVO (Figure 1). Her blood pressure was 100/70 mmHg and she had not history of any cardio-vascular disorders or diabetes.

Her laboratory findings showed pancytopenia with a WBC count of 4,680/mm^3^, RBC count of 351 × 10^4^/mm^3^, Hb level of 9.9 g/dl, Ht level of 30.3% and PLT count of 16.1 × 10^4^/mm^3^, an increased erythrocyte sedimentation rate of 54 mm/hr (<10), but APTT of 28.0 seconds was within the normal range. There was increased anti-nuclear antibody titer of x640 (<x40), ds-DNA antibody titer of 377.7 IU/ml (<12.0) and ss-DNA antibody titer of 722.4 AU/ml (<25.0) and a decline in complement levels of C_3_ 14 mg/dl (86–160), C_4_ 3 mg/dl (17–45) and CH_50_ < 10 U/ml (23–46). However, both her anti-CL IgG antibody of 9 U/ml (<10; EIA method), anti-β2 glycoprotein I antibody of 1.3 U/ml (<3.5; EIA method) and lupus anticoagulant of 1.2 (<1.4; Dilute Russell’s Viper Venom Time) were within the normal range.

Intravenous low-molecular weight heparin of reviparin was initiated, and she received steroid pulse therapy, oral fexofenadine and levocabastine eye-drops. The intraretinal hemorrhaging gradually improved, and the patient was treated with warfarin, aspirin, prednisolone and tacrolimus. Her anti-CL IgG antibody titer was examined four times every two weeks, however, all of evaluations showed negative results (2/3/1/1 U/ml). The second examination of the anti-β2 glycoprotein I antibody titer also showed a level less than 1.3 U/ml.

After informed consent was provided by the patient and parents, we examined the patient for other antiphospholipid antibodies according to the Guidelines of the European Consensus described the development of the antiphospholipid IgG antibody ELISA assay, as described previously [3]. Briefly, 1 ml of whole blood was drawn twice at an interval of 12 weeks. Microtitre plates were coated with 50 μg/ml phosphatidylcholine (PC, Nacalai tesque, Kyoto, Japan), phosphatidylethanolamine (PE, Sigma, St. Louis, USA), phosphatidylinositol (PI, Nacalai tesque, Kyoto, Japan) and phosphatidylserine (PS, ChromaDex, Inc, California, USA) and were incubated overnight. On the following day the plates were incubated with triplicate serum at a 1/10 dilution for one hour. After wash, the plates were incubated with goat anti-human IgG (Santa Cruz Biotechnology, Inc, California, USA) at a 1/2,000 dilution for one hour. Using substrate of o-phenylenediamine (Sigma, St. Louis, USA), the absorbance was read at 450 nm with ELISA reader. Age-matched three patients with APS with anti-CL IgG antibody, four patients with SLE and eight patients with non-collagen disease controls were also enrolled as disease controls. The positive cut-off value was defined as two standard deviations above the mean in the controls subjects. Since there were no positive control antiphospholipid antibodies, we calculated the optical density (OD) according to the method used in previous reports [3].

Her anti-PC IgG antibody (0.342 OD), anti-PE IgG antibody (0.190 OD), anti-PI IgG antibody (0.290 OD), and anti-PS IgG antibody (0.214 OD) were increased in comparison to not only normal controls (anti-PC; 0.095 ± 0.009 OD, anti-PE; 0.079 ± 0.019 OD, anti-PI; 0.082 ± 0.012 OD, anti-PS; 0.084 ± 0.033 OD) but also other patients with APS (anti-PC; 0.181 ± 0.076 OD, anti-PE; 0.117 ± 0.021 OD, anti-PI; 0.130 ± 0.039 OD, anti-PS; 0.129 ± 0.094 OD) and SLE (anti-PC; 0.117 ± 0.019 OD, anti-PE; 0.110 ± 0.038 OD), anti-PI; 0.097 ± 0.046 OD, anti-PS; 0.090 ± 0.037 OD). The elevation of anti-PC (0.202 OD), anti-PE (0.114 OD), anti-PI (0.181 OD), and anti-PS (0.139 OD) IgG antibodies persisted after 12 weeks.

## Conclusion

CRVO in pediatric patients is rare and has been described to be caused by cardio-vascular disorders, such as hypertension, heart failure, diabetes, atherosclerosis and APS [4]. APS may be considered present in a patient with thrombotic events and positive antiphospholipid antibodies such as anti-CL and anti-β2 glycoprotein I antibodies and lupus anti-coagulant titer that is verified on at least two occasions at least 12 weeks apart [1].

However, CL is a minor component of phospholipids in humans and anti-CL antibodies have cross-reactivity with other major components of phospholipids. Takamura *et al.*[2] described that even in both arterial and venous endothelial cells, PC (49.0 ± 0.1/50.5 ± 0.1%) and PE (28.1 ± 0.3/25.5 ± 0.4%) are more extensively distributed than CL (2.0 ± 0.1/2.3 ± 0.2%). Reshetniak *et al.*[5] found that only 40% of patients with SLE had anti-CL antibody but 60% had anti-PC antibody. Kalashnikova *et al.*[6] described that anti-PE antibody was present in 46% of anti-CL and lupus anticoagulant-negative patients.

Von Schieven *et al.*[7] found no significant difference in the prevalence of anti- anti-β2 glycoprotein I between SLE children with or without thrombosis. Berard *et al.*[8] reported that 15 patients with anti-PE antibodies without anti-CL antibodies developed thromboembolic episodes. Bertolaccini ML *et al.*[9] also showed that anti-PS/prothrombin antibodies also act as risk factor for thrombosis. Moreover, Kuksis *et al.*[10] described that decreased plasma levels of the PC is an indicator of atherosclerosis. These previous reports indicated that anti-PC, -PE, and -PS increases the risk of thrombosis and atherosclerosis even in patients without anti-CL antibody.

The examination of patients for anti-CL and anti-β2 glycoprotein I antibodies and lupus anticoagulant is insufficient to understand the essential pathology of antiphospholipid antibody-related thrombosis. Even in patients without anti-CL and anti-β2 glycoprotein I antibodies or lupus anticoagulant, there is the potential for the development of antiphospholipid antibody-related thrombosis.

### Consent

Informed consent was obtained from the patient and her parents for publication of this case report. A copy of the consent form is available for review from the Editor of this journal.

## Abbreviations

APS: Antiphospholipid antibody syndrome; SLE: Systemic lupus erythematosus; CL: Cardiolipin; CRVO: Central retinal vein occlusion; PC: Phosphatidylcholine; PE: Phosphatidylethanolamine; PI: Phosphatidylinositol; PS: Phosphatidylserine; OD: Optical density.

## Competing interests

The authors declare that they have no competing interests.

## Authors’ contributions

SK acted as the outpatient doctor for the present patient and performed the immunological examination. HG acted as the junior ward doctor for the patient. CG acted as the senior ward doctor for the patient. RO and TK acted as doctors in duty of the Department of Ophthalmology. TI is a professor in Department of Pediatrics and Child Neurology and made a significant contribution to the diagnosis and treatment of the patient. All authors read and approved the final manuscript.

## Pre-publication history

The pre-publication history for this paper can be accessed here:

http://www.biomedcentral.com/1471-2431/14/116/prepub
